# Reviewing Dengue: Still a Neglected Tropical Disease?

**DOI:** 10.1371/journal.pntd.0003632

**Published:** 2015-04-30

**Authors:** Olaf Horstick, Yesim Tozan, Annelies Wilder-Smith

**Affiliations:** 1 Institute of Public Health, University of Heidelberg, Heidelberg, Germany; 2 Steinhardt School of Culture, Education and Human Development and Global Institute of Public Health, New York University, New York, New York, United States of America; 3 Lee Kong Chian School of Medicine, Nanyang Technological University, Singapore; 4 Department of Global Health and Epidemiology, Umea University, Umea, Sweden; University of Queensland, AUSTRALIA

## Abstract

Dengue is currently listed as a “neglected tropical disease” (NTD). But is dengue still an NTD or not? Classifying dengue as an NTD may carry advantages, but is it justified? This review considers the criteria for the definition of an NTD, the current diverse lists of NTDs by different stakeholders, and the commonalities and differences of dengue with other NTDs. We also review the current research gaps and research activities and the adequacy of funding for dengue research and development (R&D) (2003–2013). NTD definitions have been developed to a higher precision since the early 2000s, with the following main features: NTDs are characterised as a) poverty related, b) endemic to the tropics and subtropics, c) lacking public health attention, d) having poor research funding and shortcomings in R&D, e) usually associated with high morbidity but low mortality, and f) often having no specific treatment available. Dengue meets most of these criteria, but not all. Although dengue predominantly affects resource-limited countries, it does not necessarily only target the poor and marginalised in those countries. Dengue increasingly attracts public health attention, and in some affected countries it is now a high profile disease. Research funding for dengue has increased exponentially in the past two decades, in particular in the area of dengue vaccine development. However, despite advances in dengue research, dengue epidemics are increasing in frequency and magnitude, and dengue is expanding to new areas. Specific treatment and a highly effective vaccine remain elusive. Major research gaps exist in the area of integrated surveillance and vector control. Hence, although dengue differs from many of the NTDs, it still meets important criteria commonly used for NTDs. The current need for increased R&D spending, shared by dengue and other NTDs, is perhaps the key reason why dengue should continue to be considered an NTD.

## Introduction

The World Health Organization (WHO) classifies dengue as a neglected tropical disease (NTD). But is dengue really neglected, and can it still be classified as an NTD? Maybe more importantly for the sake of dengue control, what is the advantage of including dengue in the list of NTDs—or is there any advantage at all?

Dengue remains largely uncontrolled globally. Prevention and control relies on vector control methods, and good case management is key to reducing mortality. Dengue is, however, expanding rapidly geographically with increased frequency and magnitude of outbreaks [1]. As a result, almost half of the world’s population lives in dengue-affected areas [2]. According to WHO, there are an estimated 50–100 million dengue infections annually, resulting in 500,000 severe dengue cases and around 25,000 dengue-attributed deaths. Newer estimates put the number of dengue infections at about 390 million each year [3]. Given these estimates, dengue has become almost as important as malaria in terms of morbidity, which is estimated to cause 207 million clinical cases [4], but dengue has a much lower mortality.

The term “NTD” emerged at the beginning of the new millennium to describe a set of diseases that were related to poverty and lacked funding for research and development (R&D) [5,6]. Dengue disease and the risk of exposure to dengue virus infection affect, however, both poor and rich populations alike. Furthermore, dengue R&D funding has significantly increased. While the bulk of R&D activities has focused on vaccine development [7], R&D funding was also earmarked for research on improved vector control with different well researched methods [8] and improved surveillance and burden of disease estimates [3,9,10], to mention only a few areas.

In this review, we develop arguments to consider if dengue is, strictly speaking, an NTD or not. For this aim we 1) review common definitions of NTDs, 2) review the lists of NTDs as defined by different agencies dealing with NTDs, 3) consider commonalities of NTDs and how dengue perhaps differs, 4) give a landscape analysis of the current research needs, reviewing ongoing areas of research, and 5) describe the changes to the dengue R&D funding over the past decade (2003–2013)—in an attempt to tackle the question, whether dengue is an NTD after all.

This is even more important in the light of the above-mentioned increased global public health importance of dengue. Because such a designation, or the lack thereof, might have implications for dengue control efforts, considering that other illnesses, such as malaria, have seen increased funding levels when treated in isolation or, as in the case of HIV and AIDS, malaria, and tuberculosis, when treated as the “big three.” This latter part will be dealt with in the discussion.

## Methods

The following methods were applied to the different parts of the review:

1. Review of common definitions of NTDs. The main objective of this part is not to present all the definitions available globally, but to show the different parts of the definitions and the process of development of the definitions over time. For the purpose of this presentation, the data collection on the definitions has been therefore limited to the process of defining NTDs as it has evolved over time at WHO and other key agencies.
2. Review of the lists of NTDs as defined by a diversity of agencies dealing with NTDs. Similar to question one, the main objective is to review criteria for the inclusion and exclusion of different NTDs by different agencies. The search is targeted and limited to key agencies, such as WHO, but also includes other key players in NTD research since their inclusion or exclusion may define different priority research areas.
3. The commonalities of NTDs and how dengue perhaps differs. This question was addressed by compiling information on the main categories of criteria describing NTDs and by putting these categories in the context of dengue in view of the existing literature.
4. A landscape analysis of the current research needs. The landscape analysis used an extensive literature review and categorised the R&D landscape using the prevention model (primary, secondary, and tertiary prevention).
5. Funding for dengue R&D. The main objective of this section is to review the existing knowledge on funding flows for dengue and other NTDs and describe mechanisms through which this funding is channelled and the type of R&D activities it supports.

## Results

### 1) Review of common definitions of NTDs

The definitions of NTDs have been developed over time and are being used by different agencies differently. One of the earliest “official” definitions of NTDs emerged from a series of WHO cohosted meetings in 2003 and 2005, the “Berlin meetings” [5,6]. NTDs are “chronic endemic tropical diseases,” “neglected in the public health arena,” and affect almost exclusively “poor and marginalised populations.” Since the establishment of the Department for the Control of Neglected Tropical Diseases in 2005, WHO has been using a similar definition, focusing on the link between poverty and NTDs and the lack of public health awareness of NTDs. “Today, neglected tropical diseases are a symptom of poverty and disadvantage. Those most affected are the poorest populations often living in remote, rural areas, urban slums and conflict zones. With little political voice, neglected tropical diseases have a low profile and status in public health priorities” [11]. In the “roadmap” to combat NTDs [12], WHO describes NTDs as “a diverse group of diseases with distinct characteristics found mainly among the poorest populations of the world,” keeping the emphasis on poverty but also noting the diversity of NTDs.

Similarly, the Centers for Disease Control (CDC) in the United States of America focuses its definition of NTDs not only on poverty but also the diversity of the diseases. CDC defines pragmatically the subgroup of diseases with effective interventions as those that can be “targeted” with consistent mass drug administration (MDA) or other effective interventions [13].

When considering the different aspects used in the definitions of NTDs and their relation to dengue (Table 1), the following can be observed: 1) poverty related − while dengue is most common in impoverished and densely populated urban areas, it equally affects rich and poor populations, 2) lack of public health attention − dengue certainly receives a lot of public health attention in many affected countries in the world, leading even to specialised national agencies for dengue control, as in Brazil and Singapore, 3) dengue is certainly—with its transmission characteristics—an endemic tropical disease, and 4) it is not unusual to find dengue in the most commonly used lists of NTDs, although these lists are otherwise very diverse.

**Table 1:** Overview of dengue and its characteristics as an NTD following the NTD criteria established by different agencies.

	Criteria											
Main criteria	Tropical Disease			Neglected needs					Neglected populations	Poverty-related		
Sub criteria	Occurrence in the tropics or subtropics	Diversity of diseases in the group of NTDs	Treatment options of NTDs (Vector control, IVM)	Neglected public health attention	R&D neglect in spending	R&D neglect in effective interventions	Causing high morbidity, low mortality	Causing Stigma and discrimination		Access to interventions	Affecting exclusively poor populations	Affecting exclusively poor countries
**1. Does dengue fulfil the different criteria for NTDs**												
**Dengue**	✓	✓	✓	No	No	✓	✓	No	No	✓	No, but more	No, but more
**2. Criteria used in main definitions for NTDs (the numbers represent the references used in the reference list of this article, Criteria not mentioned in the definitions of WHO, CDC and the Berlin meetings are taken from the literature used in this article)**												
Berlin meetings	3,4			3,4	3,4	3,4			3,4			
CDC		11	11								11	
WHO		10	12	9				.			9	

### 2) Review of the lists of NTDs as defined by a diversity of agencies dealing with NTDs

Since the definition of NTDs is very broad, the diseases included also vary for different stakeholders involved (Table 2).

**Table 2:** Lists of NTDs by different agencies.

NTD	WHO	CDC	Cochrane	PLOS NTD
Reference (see list of references)	12	11	13	14
Buruli ulcer	✓	✓		✓ *
Chagas disease	✓	✓	✓	✓ *
Cysticercosis	✓	✓	✓	✓
Dengue fever	✓	✓	✓	✓
Dracunculiasis (Guinea Worm Disease)	✓	✓		✓ *
Echinococcosis	✓	✓	✓	✓
Fascioliasis	✓	✓		✓
Human African Trypanosomiasis (African Sleeping Sickness)	✓	✓	✓	✓ *
Leishmaniasis	✓	✓	✓	✓ *
Leprosy (Hansen's disease)	✓	✓	✓	✓ *
Lymphatic filariasis	✓	✓	✓	✓ *
Onchocerciasis	✓	✓	✓	✓ *
Rabies	✓	✓		✓
Schistosomiasis	✓	✓	✓	✓ *
Soil-transmitted Helminths (STH) (Ascaris, hookworm, and whipworm)	✓	✓	✓	✓ *
Trachoma	✓	✓	✓	✓ *
Yaws	✓	✓		✓
Further included illnesses and comments	Mycetoma		Amoebiasis	More than 40 NTDs in total. Note: * Labelled “core NTDs”
	Podoconiosis		Cholera	
	Scabies		Japanese encephalitis	
	Snakebite		Leptospirosis	
	Strongyloidiasis		Paracoccidioidomycosis	
			Salmonellosis	
			Scabies	
			Shigellosis	
			Snakebite	
			Syphilis	

WHO includes 17 diseases in their list of NTDs [14]. WHO classifies these diseases according to the following criteria 1) scheduled for elimination or eradication—characterised by existing effective interventions for this purpose, 2) controllable by vector control, and 3) clinical management is key to control efforts, including early case detection and improved disease surveillance. WHO also acknowledges “other” neglected conditions, such as mycetoma, podoconiosis, scabies, snakebite, and strongyloidiasis.

CDC follows this approach and further specifies the diseases scheduled for elimination and eradication as “targeted diseases” [13].

The Cochrane library uses a different approach—also adopted by the international scientific journal *PLOS Neglected Tropical Diseases* (see below)—and includes several other NTDs: amoebiasis, cholera, Japanese encephalitis, leptospirosis, paracoccidioidomycosis, salmonellosis, scabies, shigellosis, snakebite, and syphilis [15]. However, some of the NTDs from the WHO list are not included, such as Buruli ulcer, dracunculiasis (guinea worm disease), fascioliasis, rabies, and yaws.

*PLOS Neglected Tropical Diseases* is an international open-access journal that specialises in NTDs; it classifies many more diseases as NTDs than aforementioned entities, listing more than 40 conditions [16], some of which have further subdivisions, for example, as in “haemorrhagic fevers.” The broad disease categories include helminth infections, protozoan infections, bacterial infections, viral infections, fungal infections, and ectoparasitic infections. Highlighting the link between poverty and NTDs, *PLOS Neglected Tropical Diseases* further designates 13 NTDs as “core NTDs” (Table 2). Dengue, however, is not listed in this latter group.

The arbitrary inclusion and exclusion of NTDs in the lists of different agencies dealing with NTDs makes it difficult to argue why dengue should or should not be included in the list of NTDs.

### 3) The commonalities of NTDs and how dengue differs

One of the recurrent elements in the definition of NTDs is the link to poverty (Table 1). Poverty may refer to poor access to clean water and sanitation, lack of food hygiene, or limited access to health services [12]. However, similar to NTDs, most health issues are poverty related, as shown by the Social Determinants of Health [17].

Dengue affects both rich and poor populations. However, most of the burden of disease is in very populated and impoverished urban populations. What makes other NTDs different is the fact that NTDs are almost exclusively encountered among very poor populations.

NTDs are often not prioritised in the public health agenda. Affected populations often do not have a voice, and they also do not constitute a financial market for any type of investment—in view of more market-orientated explanations. Dengue clearly differs here from other NTDs: the disease is very high on the public health agenda in many of the affected countries, especially in outbreak situations—this has been underlined by the recent warnings about potential dengue outbreaks during the FIFA World Cup 2014 [18].

Access to services by affected populations is another aspect: NTDs are often classified into three categories, related to access to services: 1) rapid-impact interventions are available; 2) the preventive power of vector control needs exploiting; and 3) improved surveillance and high-quality care in resource-limited settings play a role.

Dengue falls into categories two and three of this classification [5,6]. Vector control requires concerted efforts from several stakeholders and continuous resources in terms of funding and workforce, hence resource-limited countries are unable to implement vector control programmes at appropriate scales [19]. On the other side of the spectrum, Singapore, for example, is a country that has the resources for vector control and surveillance and is estimated to spend around US$60 million annually [20], highlighting the need for trained vector control personnel and sustained interventions similar to malaria control. Furthermore, poorer populations have difficulty in accessing health care when needed. Ease of access to care, both for prevention and health care, may be the reasons that the incidence and seroprevalence of dengue in Singapore is far lower than in its neighbouring countries [21].

Epidemiologically, NTDs are often characterised by high morbidity and low mortality. Clearly this is the case for dengue. However, many NTDs are characterised for their chronicity, causing disability and stigma—which is not the case for dengue. Dengue is a transient, short-lived infection, which may be followed by several weeks of convalescence and recovery, but it is certainly not a chronic disease that leads to disability or stigmatisation. Also, the number of deaths remain low [2,3]. Hence, the disability-adjusted life years (DALYs) lost to dengue is relatively low (Table 3).

**Table 3:** Estimated DALYs of the “Big Three” and NTDs from the Global Burden of Disease Study 2010.

Disease	DALYs (in thousands; 95% CI)
Malaria	82,685 (63,426–109,836)
HIV/AIDS	81,547 (75,003–88,367)
Tuberculosis	49,396 (40,065–56,071)
NTDs	26,060 (20,300–35,120)
Intestinal nematode infections	5,184 (2,979–8,811)
*Ascariasis*	1,315 (713–2,349)
*Trichuriasis*	638 (349–1,061)
*Hookworm Disease*	3,231 (1,695–5,732)
Leishmaniasis	3,317 (2,180–4,890)
Schistosomiasis	3,309 (1,705–6,260)
Lymphatic filariasis	2,775 (1,807–4,000)
Foodborne trematodiases	1,875 (708–4,837)
Rabies	1,462 (852–2,659)
***Dengue***	***712 (226–1*,*513)***
African trypanosomiasis	560 (76–1,766)
Chagas Disease	546 (271–1,054)
Cysticercosis	503 379–663)
Onchocerciasis	494 (360–656)
Echinococcosis	144 (69–286)
Trachoma	144 (104–189)
Yellow fever	<0.5 (0–0.5)
Other NTDs	4,724 (3,525–6,351)

DALYs = Disability-adjusted life years; NTDs = Neglected tropical diseases; CI = Confidence intervals [62].

Citation for Table 3:

Murray, CJ (2014), *Disability-adjusted life years (DALYs) for 291 diseases and injuries in 21 regions*, *1990–2010*: *a systematic analysis for the Global Burden of Disease Study 2010*. Lancet, 2014. 380(9859): p. 2197–223.

### 4) A landscape analysis of the current research needs and activities

#### Vaccine development

Despite more than 70 years of effort, a tetravalent dengue vaccine with high efficacy remains elusive. However, the current pipeline of vaccine candidates is extensive. Several vaccine development approaches have been undertaken, including live attenuated vaccines, purified inactivated vaccines, recombinant subunits, virus-like particles, and plasmid or viral vectors [22]. All these approaches are at different stages of development. The chimeric live attenuated vaccine developed by Sanofi Pasteur is the vaccine furthest in development and has now completed Phase 3 efficacy trials in Asia and South America [7,23,24]. The efficacy results have ranged from 56% to 64%, with varying degrees in efficacy for the different serotypes, age, and prevaccination flavivirus status. Although even a moderate efficacy of around 50%–60% may have a public health benefit, various issues around its limited use in flavivirus-naïve populations, low protection against serotype 2, cost-effectiveness, appropriate epidemiological thresholds for the introduction of this vaccine into national programmes, duration of protection, and long-term adverse events (or rather long-term risk for antibody dependent enhancement and more severe disease) remain to be sorted out [25]. Many unanswered questions remain before a dengue vaccine can be effectively introduced. Understanding the immune correlates for protection is now an urgent research priority. The relative suboptimal efficacy with an imbalanced low efficacy against serotype 2 results also highlights our lack of understanding of the most suitable target epitopes for vaccines. There is also an urgent need to explore the currently unknown role of nonstructural proteins, such as NS1 in dengue immunity [26,27]. In summary, the Sanofi Pasteur vaccine does show some promise [25], but this vaccine, or any other dengue vaccine for that matter, will never be a single solution to combating dengue.

#### Vector control and surveillance

Although vector control can be effective, implementation remains an obstacle [19]. Routine vector control continues to be difficult, particularly when using single interventions [28,29]. Emergency vector control is more often applied, although its effectiveness is questionable [30]. A key element in achieving more effective vector control is timely outbreak alerts generated by surveillance systems, followed by immediate vector control measures and health promotional campaigns. Dengue surveillance is, therefore, essential for detecting outbreaks and monitoring disease trends. In order to trigger timely interventions, outbreak alerts are particularly important, for both vector control and necessary reorganisation of health care delivery services, to prepare for a large surge of dengue suspected cases. However, no such evidence-based outbreak alert indicators currently exist—although studies are ongoing [9].

There is no uniform approach to surveillance, outbreak detection, and response in dengue-affected countries [9,19,31,32]. Vector surveillance and control have proven to be difficult and costly [9,19]. Another emerging problem is the development of resistance to insecticides used for vector control, such as pyrethroids and temephos [33,34]. These factors determine the need for improved monitoring and evaluation of vector control services and monitoring of insecticide resistance. Novel vector control strategies are on the horizon; and these involve releasing *Wolbachia*-infected mosquitoes or genetically engineered mosquitoes [35,36], but end-point evidence is lacking, and their routine use in national programmes may not been seen for years to come.

National dengue surveillance is done in all dengue-affected countries, except in Africa, where dengue notifications are not mandatory. “DengueNet” is WHO’s central data management system for the global epidemiological and virological surveillance of dengue fever, created in partnership with the WHO regional and country offices, Ministries of Health, WHO collaborating centres, and laboratories [37]. However, DengueNet has recently not been active. Another platform to enhance international scientific networking and exchange is the Global Health Network. The Global Health Network is a collection of websites that aim to support research by sharing knowledge and methods [38].

#### Diagnostics

For secondary prevention, early case detection and the confirmation of diagnosis are essential, with specific diagnostic tests. Although many diagnostic tests are available, most are expensive, rely on relatively complicated laboratory procedures, require consecutive tests, and often take time. A much desired improvement would be a point-of-care bedside test for early diagnosis of dengue (Rapid diagnostic test), which is quality-assured. Several such tests have been developed relying on the nonstructural protein NS1; their value is, however, still under discussion [39].

#### Case management

The key goal in tertiary prevention is to reduce case fatality. For this, timely detection of severe dengue, especially the early recognition of the development of plasma leakage and shock, is necessary so that prompt intravenous rehydration can be instituted—currently the only life-saving intervention. The WHO dengue case classification has recently been revised in order to help with this process [40]. However, there is discussion around the treatment algorithms for children and adults, the specifics for the treatment of dengue in patients with comorbidities and during pregnancy, as well as the best type of fluids to use [41]. Clinical case definitions and clinical management show a high degree of heterogeneity between countries, which underscores the urgent need for well-designed prospective clinical trials [42,43]. Studies on early warning symptoms and signs are now ongoing [10].

Specific antiviral drugs for the treatment of dengue have not yet been discovered. Several strategies have been pursued to identify inhibitors of DENV through targeting both viral and host proteins including the repurposing hepatitis C virus inhibitors for DENV. Along the developmental process, many drug candidates were discovered, but they did not advance beyond the stage of hit-to-lead optimisation due to their poor selectivity or pharmacokinetic properties [26]. Dengue antiviral patents have doubled, if not increased exponentially in the past 15 years [44].

#### Dengue economics

Dengue costing studies and cost-effectiveness of dengue interventions are relatively sparse, and results have often been conflicting because of information gaps [45]. How do we prioritise research, health policy, and financial resources toward dengue control? We need to move away from narrowly defined benefit categories, such as health care cost savings, care-related productivity gains, and health gains in reduction in morbidity and mortality and consider outcome-related productivity gains and community economic externalities [46]. Outbreak control spending, income from tourism, and long-term economic productivity are important factors to consider in economic evaluations of dengue disease and potential future vaccination implementation [46].

### 5) Funding for dengue R&D

The rapid geographical expansion of dengue [1,47] has been paralleled by an increase in dengue research and research funding. This increase was further spurred by the perceived threat of dengue to currently noninfected areas, including the threat to Western countries. Imported dengue cases via international travellers to Western countries are rising [48]. Another important factor that has led to more spending in dengue research is the fact that many of the currently dengue endemic countries have experienced rapid economic growth and emerged as new strong markets—on the other hand, this has also resulted in fewer grants from funding agencies orientated towards poorer countries. Dengue has also attracted the attention of novel actors in the field from both the academic and corporate world. The most active (i.e., having a significant number of dengue research projects funded in the last ten years) and large-scale funding agencies are the Bill and Melinda Gates Foundation, the National Institute of Health (United States of America), the Wellcome Trust (United Kingdom), and the European Union (EU), underlining the fact that the vast majority of funding dengue research centres receive remains in nondengue endemic countries, both at the academic and corporate level, with the notable exceptions of Brazil and Singapore. The European Commission (EC) has been supporting research programmes in infectious diseases since the 1990s. A total of 25 projects on dengue have been supported [49]. In 2010, the EC launched a call under the Seventh Framework Programme with the title of “Comprehensive control of dengue fever under changing climatic conditions” [50]. The EC awarded a total of approximately €18 million to three consortia [10,51]. The consortia comprise 38 partners from around the world.

To combat NTDs, the Novartis Institute of Tropical Diseases (NITD) [44] was founded in 2002 through private public funding from Novartis and the Singapore Economic Development Board. One of NITD's missions is to develop antivirals for dengue virus. The International Consortium on Antivirals (ICAV) is a not-for-profit drug development organisation whose mandate is also to discover and develop affordable antiviral therapies for NTDs [52]. ICAV operates through a network of collaborative laboratories and by liaising with governments and other stakeholders. The most recent large-scale funding on dengue antiviral drug discovery and design is from the FP7-SILVER project, which has dengue as one out of its three main targets for antivirals.

An empirical analysis of official development assistance (ODA) for health during 2003–2007 showed that on average only 0.6% of annual health ODA supported NTD control programmes, in comparison to the “big three” HIV and AIDS, malaria, and tuberculosis programmes, which had average shares of 36%, 3.6%, and 2.2%, respectively [53]. This does not correspond to the burden of disease caused by NTDs (Table 3). However, when comparing the global burden of NTDs in DALYs, dengue appears to be well funded compared to other NTDs because dengue has a relatively low mortality rate.

While the 2013 World Health Assembly resolution on NTDs urged member states to take ownership of national programmes and international partners to provide sufficient and predictable funding streams for implementation, R&D of new tools, and technologies, it also highlighted the growing threat and spread of dengue in affected countries and beyond [54]. Dengue is not one of the ten NTDs prioritised in the 2012 London Declaration. Against a background of increasing ODA for health in general and national NTD programmes in particular, the financial support for dengue prevention and control programmes in endemic countries has been strictly limited to isolated outbreak response through emergency funds [55]. Dengue endemic countries rely primarily on their own national resources for dengue control and surveillance activities.

A six-year review of global funding for neglected disease R&D commissioned by the Bill and Melinda Gates Foundation showed that total R&D funding increased by just about US$600 Million, from 2.6 Billion US $ to 3.2 Billion US $, during 2007–2012 (Table 4) [56,57]. There is widespread agreement on the need for far more investment into R&D of drugs, diagnostics, and vaccines to have a major impact on the global burden of NTDs [58,59,60]. Most notable during this period was, however, the significantly increasing share of dengue R&D funding from 3.2% to 7.9%. In 2012, dengue was the fourth highest funded NTD globally following HIV and AIDS, malaria, and tuberculosis, with an investment of nearly a quarter billion US $ (Table 4). Linking R&D investments to disease burden estimates (as measured in DALYs), it was argued that dengue attracted far more R&D investment given its burden relative to other NTDs because dengue, unlike several other NTDs, has a traditional commercial market as it also affects the populations in middle- and high-income countries [59]. However, the global public health burden of dengue infections in 2010 has been put at 390 million infections [3], which is about four times higher than previous estimates used by WHO [61]. A major challenge in estimating dengue disease burden has been the temporal and geographic variation in dengue incidence and mortality rates, which introduces large prediction errors into commonly used models [3,62].

**Table 4:** Global neglected disease R&D funding by disease, 2007–2012* (US $, millions; percentage of total funding, %).

Disease	2007	2008	2009	2010	2011	2012	Disease total funding
HIV/AIDS	1,083 (42.3)	1,165 (39.4)	1,139 (35.9)	1,073 (35.0)	1,029 (33.8)	1,064 (33.6)	6,553 (36.5)
Malaria	468.4 (18.3)	541.7 (18.3)	593.9 (18.7)	547 (17.9)	558.8 (18.4)	542.5 (17.1)	3,252.3 (18.1)
Tuberculosis	410.4 (16.0)	445.9 (15.1)	550.9 (17.4)	575.4 (18.8)	525.8 (17.3)	502.1 (15.9)	3,010.5 (16.8)
Dengue	***82 (3*.*2)***	***126*.*8 (4*.*3)***	***165*.*8 (5*.*2)***	***177*.*6 (5*.*8)***	***229 (7*.*5)***	***248*.*9 (7*.*9)***	***1*,*030*.*1 (5*.*7)***
Diarrhoeal diseases	113.9 (4.4)	132.2 (4.5)	180.4 (5.7)	158.9 (5.2)	152.2 (5.0)	152.2 (4.8)	889.8 (5.0)
Kinetoplastids	125.1 (4.9)	139.2 (4.7)	162.3 (5.1)	147.9 (4.8)	131.7 (4.3)	136.3 (4.3)	842.5 (4.7)
Bacterial pneumonia & meningitis	32.5 (1.3)	90.8 (3.1)	69 (2.2)	92.9 (3.0)	96.6 (3.2)	99.2 (3.1)	481 (2.7)
Helminths (worms and flukes)	51.6 (2.0)	66.8 (2.3)	79.4 (2.5)	73.7 (2.4)	81.1 (2.7)	84.4 (2.7)	437 (2.4)
Salmonella infections	9.1 (0.4)	39.5 (1.3)	39.4 (1.2)	44 (1.4)	44.4 (1.5)	52.6 (1.7)	229 (1.3)
Leprosy	5.6 (0.2)	9.8 (0.3)	11 (0.3)	8.8(0.3)	7.4 (0.2)	13.1 (0.4)	55.7 (0.3)
Trachoma	1.7 (0.1)	2.1 (0.1)	1.8 (0.1)	4.5 (0.1)	9.6 (0.3)	8.7 (0.3)	28.4 (0.2)
Buruli ulcer	2.4 (0.1)	2 (0.1)	1.8 (0.1)	5.5 (0.2)	5.8 (0.2)	6.1 (0.2)	23.6 (0.1)
Rheumatic fever	1.7 (0.1)	2.2 (0.1)	3 (0.1)	1.7 (0.1)	0.8 (0.03)	0.9 (0.03)	10.3 (0.1)
Platform technologies	10 (0.4)	16.3 (0.6)	22.1 (0.7)	27.4 (0.9)	17.2 (0.6)	43.8 (1.4)	136.8 (0.8)
Core funding of a multi-disease R&D organisation	110.9 (4.3)	101.1 (3.4)	74.1 (2.3)	76.9 (2.5)	91.3(3.0)	109.6 (3.5)	563.9 (3.1)
Unspecified disease	51.6 (2.0)	74.7 (2.5)	75.7 (2.4)	47.5 (1.6)	64.7 (2.1)	100.3 (3.2)	414.5 (2.3)
Total funding	***2*,*560 (100)***	***2*,*956 (100)***	***3*,*170 (100)***	***3*,*063 (100)***	***3*,*045 (100)***	***3*,*165 (100)***	***17*,*958 (100)***

Global NTD research and development funding tabulated by disease, using data from G-Finder public search facility, adjusting 2008–2012 funding data for inflation and reporting in 2007 US $.

*2008–2012 funding data has been adjusted for inflation and is reported in 2007 US dollars (US $).

Source: Data compiled from the G-FINDER public search facility: https://gfinder.policycures.org/PublicSearchTool/.

**Fig 1:**
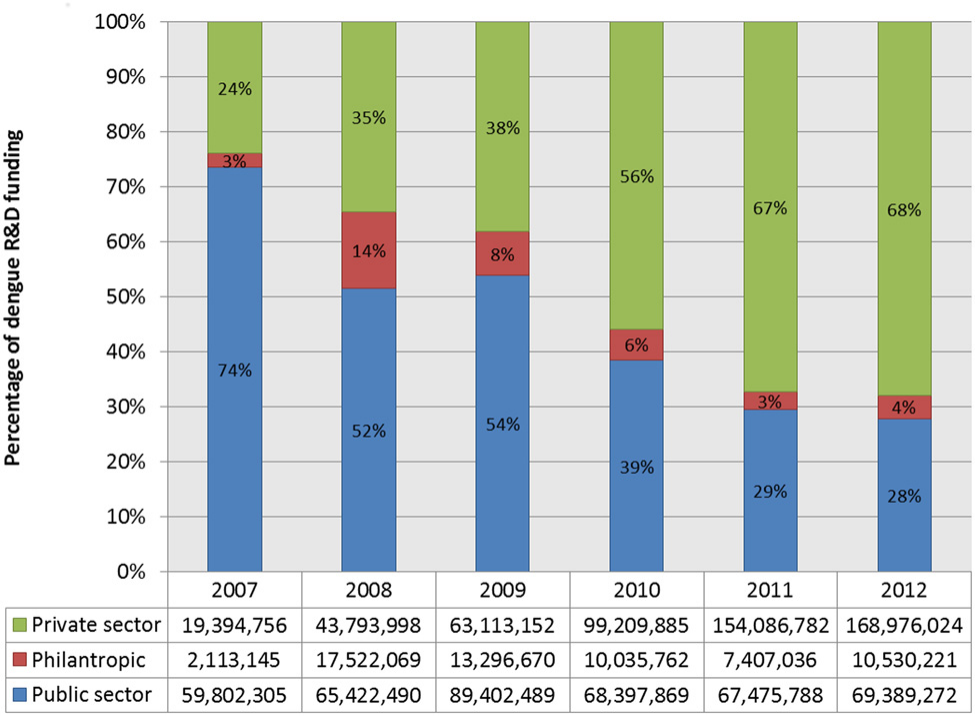
Dengue R&D funding by type of funder, 2007–2012 (US $). Global dengue research and development funding tabulated by type of funder, private, philanthropic, or public funding, using data from G-Finder public search facility, adjusting 2008–2012 funding data for inflation and reporting in 2007 US $. https://gfinder.policycures.org/PublicSearchTool/.

Global funding for neglected disease R&D has been primarily driven by the public sector during 2007–2012, although funding from high-income governments has been worryingly flat or down in recent years [57]. While this funding pattern held true across the board for most NTDs, the pharmaceutical industry was a mainstay of dengue R&D funding throughout this period (Fig 1) [56]. Most notable in this period was, however, the shifting landscape of dengue R&D funding from the public sector to the pharmaceutical industry. In 2012, about 68% of dengue R&D funding came from the pharmaceutical industry—with most of this funding probably allocated for dengue vaccine development—whereas its share was 24% in 2007 (Fig 1) [56]. Overall, the pharmaceutical industry provided more than 50% of total dengue R&D funding, investing about US$550 million over six years. As with most other NTDs, the US government and the Bill and Melinda Gates Foundation are the other mainstays of dengue R&D funding during this period (Table 5) [56]. In particular, the US National Institutes of Health accounted for a quarter of total dengue R&D funding. Notable among the top ten funders is the government of Brazil with its US$32 million contribution.

**Table 5:** Top ten funders of dengue R&D, 2007–2012* (US $, millions; percentage of total funding, %).

	Funder	2007	2008	2009	2010	2011	2012	Funder total
1	Aggregate industry	19.4 (24)	43.8 (35)	63.1 (38)	99.2 (56)	154.1 (67)	169.0 (68)	548.6 (53)
2	US NIH	34.6 (42)	26.6 (21)	54.0 (33)	46.3 (26)	51.4 (22)	47.3 (19)	260.3 (25)
3	US DOD	14.4 (18)	7.5 (6)	10.5 (6)	5.5 (3)	4.0 (2)	4.3 (2)	46.1 (4)
4	Gates Foundation	1.0 (1)	16.3 (13)	11.7 (7)	6.5 (4)	0.1 (<1)	4.6 (2)	40.2 (4)
5	Brazilian Government	3.9 (5)	14.1 (11)	11.0 (7)	1.5 (1)	0.2 (<1)	1.3 (1)	32.1 (3)
6	The Wellcome Trust	1.0 (1)	1.2 (1)	1.4 (1)	1.8 (1)	4.0 (2)	4.6 (2)	14.0 (1)
7	Institute Pasteur	2.0 (2)	1.7 (1)	2.0 (1)	2.4 (1)	1.7 (1)	1.9 (1)	11.8 (1)
8	Australian government	0.7 (1)	3.9 (3)	1.3 (1)	3.1 (2)	2.0 (1)	2.8 (1)	13.8 (1)
9	European Commission	1.6 (2)	1.3 (1)	1.1 (1)	0.5 (<1)	0.5 (<1)	1.3 (1)	6.3 (<1)
10	US CDC	0.0 (0)	0.0 (0)	1.4 (1)	1.4 (1)	0.0 (0)	2.3 (1)	5.1 (<1)
	Sub-total top 10 funders	78.7 (96)	116.6 (92)	157.6 (95)	168.0 (95)	217.9 (95)	239.5 (96)	978.2 (95)
	Dengue total funding	82.0	126.8	165.8	177.6	229.0	248.9	1,030.1

Global dengue research and development funding tabulated by main funding agency—top ten funders, using data from G-Finder public search facility, adjusting 2008–2012 funding data for inflation and reporting in 2007 US $.

*2008–2012 funding data has been adjusted for inflation and is reported in 2007 US dollars (US$).

Source: Data compiled from the G-FINDER public search facility: https://gfinder.policycures.org/PublicSearchTool/.

**Fig 2:**
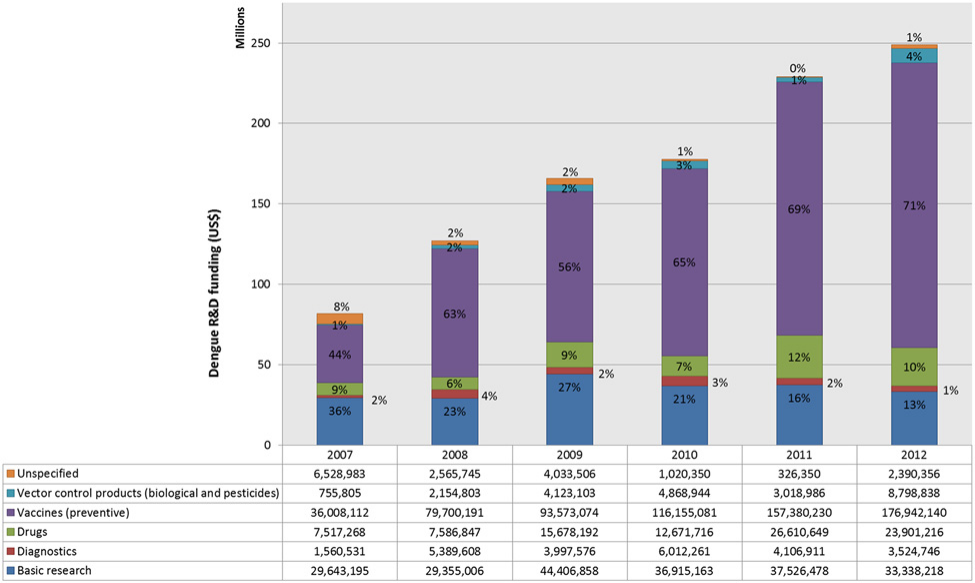
Dengue R&D funding by product area, 2007–2012 (US $). Global dengue research and development funding tabulated by product area, basic research, diagnostics, drugs, vaccines, vector control products, and unspecified, using data from G-Finder public search facility, adjusting 2008–2012 funding data for inflation and reporting in 2007 US $. https://gfinder.policycures.org/PublicSearchTool/.

A closer examination of dengue R&D funding also revealed that there are marked differences in investments across different dengue products during this period (Fig 2) [56]. Each year, the vast majority of dengue R&D funding went to vaccine development, followed by basic research. The pharmaceutical industry invested over 90% of its outlay into vaccine development, which corresponds to nearly 77% of total investment into dengue vaccines (data not shown). Sanofi Pasteur has spent more than €1.2 billion on developing its vaccine candidate and setting up manufacturing capacity for example.

While funding for drug development, mainly driven by the public sector, has increased over this period, investments into dengue diagnostics and vector control products remained well below the level needed to develop any novel products within a reasonable timeframe. The drug development pipeline for dengue currently includes only multiple early stage drug candidates [63]. There is a need for a validated and inexpensive rapid diagnostic test for early diagnosis and clinical management of dengue [39]. Dengue is not in the product development portfolios of the Drugs for Neglected Diseases Initiative (DNDi) or the Foundation for New Innovative Diagnostics (FIND).

## Discussion/Conclusion

Several shared characteristics of dengue with other NTDs support its classification as an NTD: dengue is a disease of tropical and subtropical countries with limited resources. Although the disease does not only affect poor populations, dengue is nevertheless most common in impoverished and densely populated urban areas and has clear poverty-related aspects where access to prevention through vector control and high quality medical care might be limited. Also, resource-limited populations tend to be affected more often at not only the micro (individual) but also the macro level (comparison of adjacent countries). Furthermore, dengue is characterised by high morbidity and low mortality, similar to other NTDs. Dengue, similar to most NTDs, currently lacks effective community-based interventions, especially for primary prevention, and more R&D is urgently needed.

On the other hand, dengue differs from many of the other NTDs: it is an acute short-lived disease and it does not cause chronic disability, stigma, or discrimination. Unlike many other NTDs, it is also not a disease that is “hidden” or remains unnoticed by the public. On the contrary, dengue is publicly a highly visible disease, frequently makes it to the front pages of national newspapers, is often a theme during political elections, and receives more funding than other NTDs at the national level. Furthermore, dengue R&D funding has increased exponentially in the past decades, particularly for dengue vaccine development.

The question then remains why it would be useful to keep dengue in the group of NTDs, or whether the approach of the “big three: HIV and AIDS/malaria/tuberculosis” with a “global fund” funding system would be more appropriate for dengue. From the viewpoint of the authors, the main difference between the “big three” and dengue is that there is insufficient R&D spending for dengue—as outlined above—and the main problem is not increased funding to implement proven and cost-effective interventions in resource-poor settings, as in the case of the “big three.” For dengue, there is currently insufficient evidence on currently available interventions to recommend large-scale implementation and warrant such an “implementation funding” mechanism. The key factor for dengue is the need for increased R&D spending, shared by dengue and other NTDs, and this is perhaps the key reason why dengue should be considered an NTD.

In view of the need for further R&D, the question remains then whether a recommendation for future R&D spending can be made. It is difficult to make specific recommendations on the level of funding needed or the best delivery of funding based on this analysis; however, the following conclusions emerge: 1) dengue vaccine R&D will probably continue, and the funding will come mainly from the industry, 2) the search for an optimal vaccine should not stop current vector control efforts, and the focus should be on integrating more effective vector control measures with vaccine introduction, 3) R&D for dengue diagnostics and drugs, as well as 4) further clinical research, and 5) research for improved integrated surveillance are needed, 6) there may be opportunities to integrate dengue prevention and community mobilisation activities with other NTDs, where mass drug administration and community involvement are implemented, and 7) a new priority setting exercise, similar to what was done in 2005 by the WHO/TDR Dengue Scientific Working Group, is overdue [64].

**Key Learning Points**

- Dengue meets many of the criteria commonly used for NTDs, although research spending has increased notably in the past decade, primarily for vaccine development.
- Increased research spending has not led to groundbreaking tools or interventions for improved dengue control from a public health perspective, although vaccine development has been partially successful.
- Further increase in research spending is warranted to achieve improved dengue control, perhaps combining existing (vector control) and new (vaccine) interventions with operational and implementation research, while focusing on basic research (drug development and new vector control methods), clinical research, and research on integrated surveillance.
- The current need for increased research spending, shared by dengue and other NTDs, is perhaps the key advantage, why dengue should be considered as an NTD.

**Top Five Papers**

1. Bhatt S, Gething PW, Brady OJ, Messina JP, Farlow AW et al. (2013) The global distribution and burden of dengue, Nature 2013, Volume 496, pp 504–507.
2. Horstick O, Morrison AC (2014) Dengue Disease Surveillance: Improving Data for Dengue Control. PLoS Negl Trop Dis 8(11): e3311.
3. Wilder-Smith A (2014) Dengue Vaccines: dawning at last. Lancet Infectious Dieseases. The Lancet, Early Online Publication, 11 July 2014, doi: 10.1016/S0140-6736(14)61142-9 http://www.thelancet.com/journals/lancet/article/PIIS0140-6736%2814%2961142-9/fulltext?_eventId=login
4. WHO (2006) Report of the Scientific Working Group on Dengue, WHO, Geneva, 01–05 October 2006, http://www.who.int/tdr/publications/tdr-research-publications/swg-report-dengue/en/index.html
5. WHO (2012) Global Strategy for dengue prevention and control 2012–2020, http://apps.who.int/iris/bitstream/10665/75303/1/9789241504034_eng.pdf
